# Towards Child-Appropriate Virtual Acoustic Environments: A Database of High-Resolution HRTF Measurements and 3D-Scans of Children

**DOI:** 10.3390/ijerph19010324

**Published:** 2021-12-29

**Authors:** Hark Simon Braren, Janina Fels

**Affiliations:** Institute for Hearing Technology and Acoustics, RWTH Aachen University, Kopernikusstraße 5, 52074 Aachen, Germany; Janina.Fels@akustik.rwth-aachen.de

**Keywords:** head-related transfer function, spatial hearing, children, binaural technology, 3D-scan, HRTF database

## Abstract

Head-related transfer functions (HRTFs) play a significant role in modern acoustic experiment designs in the auralization of 3-dimensional virtual acoustic environments. This technique enables us to create close to real-life situations including room-acoustic effects, background noise and multiple sources in a controlled laboratory environment. While adult HRTF databases are widely available to the research community, datasets of children are not. To fill this gap, children aged 5–10 years old were recruited among 1st and 2nd year primary school children in Aachen, Germany. Their HRTFs were measured in the hemi-anechoic chamber with a 5-degree × 5-degree resolution. Special care was taken to reduce artifacts from motion during the measurements by means of fast measurement routines. To complement the HRTF measurements with the anthropometric data needed for individualization methods, a high-resolution 3D-scan of the head and upper torso of each participant was recorded. The HRTF measurement took around 3 min. The children’s head movement during that time was larger compared to adult participants in comparable experiments but was generally kept within 5 degrees of rotary and 1 cm of translatory motion. Adult participants only exhibit this range of motion in longer duration measurements. A comparison of the HRTF measurements to the KEMAR artificial head shows that it is not representative of an average child HRTF. Difference can be seen in both the spectrum and in the interaural time delay (ITD) with differences of 70 μs on average and a maximum difference of 138 μs. For both spectrum and ITD, the KEMAR more closely resembles the 95th percentile of range of children’s data. This warrants a closer look at using child specific HRTFs in the binaural presentation of virtual acoustic environments in the future.

## 1. Introduction

Real-world acoustic environments are highly complex. The soundfield in a given room is defined by, among other factors, the sound sources in and outside the room together with transmission and absorption characteristics of the room’s surfaces, all contributing to the auditory signal received by an observer in said environment. For example, in a classroom these factors affect both the speaking behavior of the teacher [1] as well as the speech intelligibility and learning abilities of the students [2,3], (for a review, [4]). To analyze what room parameters cause these effects, objective metrics, such as speech transmission index (STI), signal-to-noise ratio (SNR) or reverberation time (RT), give a description of the soundfield and room characteristics as they were at the time of a given recording or experiment. These metrics only allow a-posteriori analyses, with limited control over the underlying acoustic parameters.

Modern simulation and auralization techniques [5] allow the recreation of such complex sound fields with a high level of control over the objective metrics. Room acoustics can be fine tuned through the adaptation of size and surface properties of virtual rooms, the acoustic signals can be adjusted through the positioning and control of static and dynamic virtual sound sources. The last step in the auralization chain is the presentation of the simulated sound field using a suitable method, which should be able to reproduce the three-dimensional nature of such sound fields. Loudspeaker based reproduction methods such as ambisonics [6,7] or VBAP [8] require a significant number of loudspeakers and in theory an acoustic free-field environment. Hence, headphone based reproduction using the binaural method [9,10] is often the method of choice in listening experiments, in part due to reduced hardware requirements while creating a highly plausible and authentic virtual acoustic environment [11,12].

To recreate the sound pressure at the ear drum as it would be in a given environment though headphones, a suitable, generally open-back pair [13] should be used and the headphone transfer function needs to be compensated through individual measurements [14]. The spatial information is provided through the head-related transfer function (HRTF) used in the binaural presentation. It contains the different temporal and spectral cues interpreted by the auditory system to localize a sound source in 3D-space [9,15,16]. These cues are the result of reflections and refraction of an incoming sound-wave at the head, torso, and pinnae. The individual size and shape of these anthropometric features differ greatly between humans, thus the resulting HRTFs are highly individual. HRTF measurements require specialized equipment and processing techniques [17,18,19], (for a review, [20]) that are not commonly available. Instead, existing HRTF datasets from artificial heads, such as the KEMAR [21,22], are often used in practice.

From experiments with adults, it is known that the use of non-individual HRTFs, i.e., the participants listen through someone else’s ears, comes with perceptual side-effects such as localization inaccuracies or front-back confusion [23,24,25]. This is due to a mismatch of the spatial cues from the foreign head and ears, which would first need to be learned to not affect the performance [26]. Oberem and colleagues showed that the use of non-individual HRTFs with untrained participants affected their performance in complex auditory environments [27]. Jakien and colleagues [28] found, that non-individual HRTFs reduced the speech intelligibility in virtual environments, which was later confirmed through listening experiments [29] and through established auditory models [30].

While these results are the result of experiments with adult participants, similar results can be expected for children. Children’s anthropometric features are still growing well into their teen years [31,32]. Hence, their HRTFs differ from adult or standardized artificial heads [33] HRTFs based on adult geometries. Nonetheless, the same artificial head HRTFs are used in virtual environments for children (e.g., [34,35,36]). Kids under the age of 10 are especially of interest here, as the growth-rate of the pinnae significantly decreases around that age [31]. Perceptual evaluations of the effect of non-individual HRTFs with children have, to the knowledge of the authors, not yet been reported. Objective metrics from room acoustics simulations [37] and classroom measurements [38,39] using a simplified artificial head for children [40] found differences especially in speech intelligibility.

One alternative towards an improved binaural experience is the individualization of the used HRTF [41,42,43,44], (for a review, [45]). Based on existing databases of HRTF measurements and anthropometric dimensions of the head and pinna, the temporal and spectral features of an HRTF are adapted to better match the listener’s own, reducing the above mentioned negative effects. While adult HRTF databases (e.g., [46,47,48]) are widely available, HRTF measurements of children are not. As a potential alternative, the use of 3D-models head and torso models of children to calculate individual HRTFs has been discussed the past [40,49]. Datasets of models needed for the calculations are, however, only available from a small number of children [49] or as an highly abstracted average head shape [40] that was validated through acoustic measurements up to 8 kHz. The datasets presented in this publication combine both acoustic HRTF measurements and high-resolution 3D models of currently 23 children as part of an openly available database, with the intention of adding more in the future to create a database more representative of the diversity among children.

## 2. Materials and Methods

### 2.1. Participants

Twenty-six children (ages 5–10 year, mean 7.3 year), consisting of 8 male and 17 females, participated in the study. An additional dataset of one three-year old is included in the CHASAR database but was excluded from further evaluations presented in this paper. The participants were recruited from 1st- and 2nd-year school children and their siblings in the same age group. The recruitment took place in a local school in Aachen, Germany, with consent of the headmaster. In the study, the children were accompanied by a parent or guardian in groups of one or two. Informed consent was given for the publication of the measurement and 3D-Scan. Given the choice to consent to publication the original 3D-scan or an anonymized version, 21 of the 23 participants only gave consent to an anonymized publication. The acoustic implications of the anonymization is further discussed in Section 3.6 The children received a 10 EUR voucher for a local bookstore as compensation for their participation.

### 2.2. HRTF Measurement

**Figure 1:**
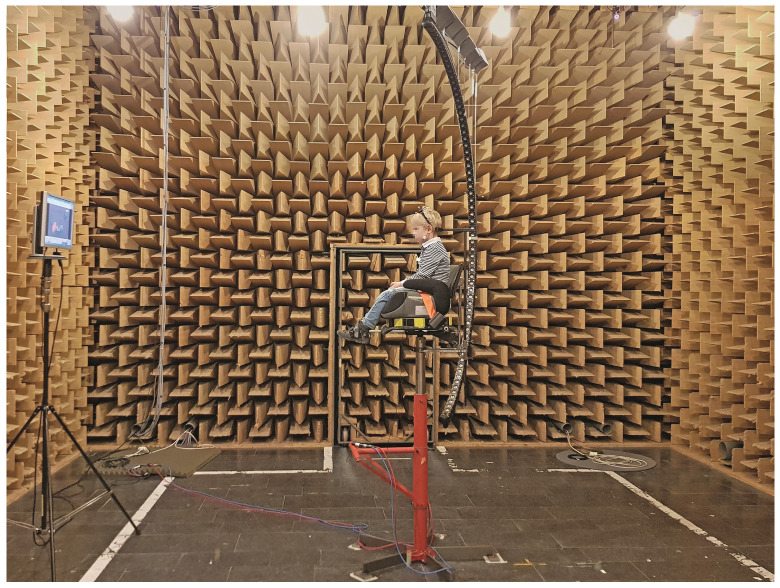
Child during the fast HRTF measurement sitting in measurement system consisting of a 64-loudspeaker arc, a height adjustable chair and monitor in front of the participant.

The HRTF measurement took place in the hemi-anechoic chamber at the Institute for Hearing Technology and Acoustics at RWTH Aachen University. The room is equipped with 80 cm mineral wool absorbers mounted to all walls and the ceiling, providing free-field conditions down to 100 Hz. The floor constitutes a rigid, reflecting boundary that was considered in post-processing by cutting off the floor reflections by time-windowing the measured impulse responses. This allowed the placement of additional equipment in the room, as long as their reflection paths were longer than the first floor-reflection, i.e., the reflection from the equipment were cut off by the same time window. The continuous HRTF measurement system [18,50] was used to acquire 5 × 5-degree (azimuth × elevation) resolution HRTF datasets. During the measurement, an arc containing 64 Tang Band W1-2025SA loudspeakers (Tang Band Speaker, Taipei, Taiwan) was continuously rotated around the participant at a constant angular velocity. It was actuated by a silent stepper motor mounted above the participant. The motor is attached to mounting rods protruding through the ceiling absorbers and controlled by the ITA-Toolbox [51] measurement software implemented in MATLAB. The frequency response of the loudspeaker is relatively flat between approximately 500 Hz to 18 kHz [52]. At low frequencies, the HRTF can be considered flat and linear in phase [53] and is interpolated by frequency domain interpolation towards unity gain at 0 Hz. The participants were sitting on a height-adjustable chair with their ear canal entrances and thereby their head-related coordinate system aligned with the center of the sphere-cap circumscribed by the rotating arc. The head-related coordinate system is defined as a (right-handed) Cartesian coordinate system with the *y*-axis going through the ear canal entrances with the origin in the center between the two ears. The *z*-direction points straight up and *x* points forward in the direction of the participant’s gaze. The children’s feet were placed on a footrest when needed, to ensure that the dangling feet would not impede the loudspeaker arc when it moves in front of the participant. The measurement system can be seen in Figure 1.

Two Sennheiser KE3 microphones (Sennheiser electronic GmbH & Co. KG, Hannover, Germany) in closed silicone hearing-aid-domes were placed in the ear-canal entrances of the participants. The hearing-aid-dome size was adequately chosen to guarantee a good fit for each participant, sealing the ear canal entrance. Interleaved swept sine signals [54] were used as measurement signals. The use of these signals allows multiple loudspeakers to be active at the same time, while allowing a clean separation of the individual impulse responses in post-processing. This shortens the overall measurement time for a given angular resolution. A full 360 degree of the arc rotation and thus one measurement was performed in about 3 min. During the HRTF measurement, a parent or guardian was allowed in the anechoic chamber. They were instructed to stay silent and stand far enough away so as not to interfere with the HRTF measurement. The children were instructed to watch an age-appropriate, silent TV show on a screen positioned at head height in front of them. This led to a decreased head movement as they could focus on the TV screen instead of being tempted to look around during the measurement. In addition, the head rested against an adjustable headrest and was tracked using an optical 6 degrees of freedom (DOF) tracking system by Optitrack (Natural-Point Optitrack, Corvallis, OR, USA). For this purpose, the children wore a hairband with an attached tracking body on their head. The system was calibrated to the head-related coordinate system of each participant before each measurement. For this calibration, the positions of the ear canal entrance relative to the rigid body on the head were measured using two tracked marker-pens. From these known positions the center of their interaural axis was calculated and its movement was tracked throughout the measurement. The measurement including the adjustments of the chair and the optical calibration and setup took about 15 min.

### 2.3. HRTF Processing

To calculate the HRTF from the above-described measurements, a reference measurement using the same microphones was recorded. The microphones were positioned in a microphone stand in the center of the arc. A transfer function from each loudspeaker was recorded, capturing the influence of the microphone and each loudspeaker. In a first step, the measurements from the in-ear microphones were split into separate, direction-specific impulse responses. Each was referenced to the loudspeaker corresponding to their elevation. The unwanted reflections from the floor and equipment in the measurement room were cut off using a Hann-shaped time window with a fall time of 2 ms leaving only the actual HRTF with a length of 368 samples (i.e., 7.67 ms at 48 kHz sampling rate). At this stage, each impulse response was assigned the position of their respective loudspeaker at the start of the sweep signal from which the impulse response was calculated. In a next step the frequency-dependent position caused by the continuous rotation of the loudspeaker during the swept sine excitation was considered as described by [50]. The exact position of each loudspeaker was calculated from the rotational speed of the arc for each transfer function and each frequency bin separately. In a frequency-dependent spherical harmonics (SH) decomposition with a maximum order of 75 [52], these per-frequency-bin loudspeaker positions were considered and an HRTF on an equiangular 5-degree × 5-degree grid was calculated from the SH-coefficients.

### 2.4. 3D-Scan

After the HRTF measurement, a high-resolution 3D-model of each participant was captured using an Artec Space-Spider 3D-scanner (Artec 3D, Luxembourg). The 3D-scanner works by projecting a line pattern onto the scanned surface and capturing a series of images of the distorted lines at up to 8 frames per second. From these images, a point cloud of the surface was extracted by aligning and combining multiple scans, resulting in a 3D-surface model with sub-millimeter resolution. Since this scanning method has problems with diffusely reflecting surfaces (such as hair), a wig-cap with visual markers was placed on the participants head for the duration of the scan. This improves the scanning process by providing a smooth surface with better reflection properties and helps in post-processing where the visual markers can be used to better align subsequent scans. To scan a whole head and upper torso, 16–20 separate scans were recorded. Each ear was scanned from a minimum of five directions, to ensure an accurate ear-model and account for the complex geometry of the pinna. The 3D-scan took approximately 25 min per participant. During that time, the children watched a child-appropriate TV-show helping them keep their posture steady and not make sudden movements which cause the scanner to lose its tracking and resulting in a reduced scan quality.

### 2.5. Mes Processing

The 3D-processing was mainly performed in Artec Studio 13. Here the scans were aligned and turned into 3D-surface meshes. The resulting meshes were too detailed to be used e.g., in simulations. Hence, they were simplified in Artec Studio using the built-in smoothing and mesh simplification routine (settings: error: 0.01, max neighb normal angle: 120). Remaining artifacts (e.g., holes in areas missed in the scanning procedure or superfluous detail from clothes and unwanted hair sticking out under the cap) and further simplifications were handled in Autodesk Meshmixer (Autodesk Inc., San Rafael, CA, USA). The models were then aligned to the head-related coordinate system. For publication, the 3D-models were anonymized by means of aggressive surface smoothing of the face in Meshmixer to remove identifiable facial details while maintaining detailed models, especially of the pinna geometry. The effect of the anonymization on simulated HRTFs is evaluated in Section 3.6.

### 2.6. HRTF Simulation

The boundary element method (BEM) is a well-established method for simulating HRTFs based on 3D-surface meshes of the head and upper torso [55,56]. It can be used to calculate high-resolution HRTF datasets using the reciprocity principle, i.e., exchanging the in-ear microphones by excitations, and evaluating the resulting sound field at the locations where the real-world loudspeakers would be located. The simulated HRTFs presented in this study were calculated using the mesh2hrtf simulation tool version 0.4.0 [57,58] and in part validated using Comsol Multiphysics (Comsol AB, Stockholm, Sweden). Comsol’s meshing algorithm was used to create lower-resolution simulation meshes from the high-resolution 3D-scans to reduce computational effort and ensure simulation accuracy. The meshes were reduced based on a maximum simulation frequency of 20 kHz to a maximum edge length of 3.7 mm. Additional settings in Comsol’s meshing algorithm, namely the resolution in narrow regions (set to 0.7) and curvature factor (set to 0.3), increased the mesh resolution in non-flat regions of the geometry to retain an accurate representation of the 3D-shape, especially of the pinnae. The resulting simulation meshes consist of approximately 35,000–42,000 nodes, depending on the physical size of the head and torso which the mesh resembles as well as the surface complexity of the mesh.

### 2.7. HRTF Distance Metrics

An adequate distance metric that takes the spatial and frequency dependence of the transfer functions into account was needed to compare HRTFs. For this study, the inter-subject spectral differences (ISSD), mentioned by [44], was chosen as a single digit metric to compare two HRTF datasets. The ISSD for each direction (denoted by its elevation angle θ and azimuth angle ϕ) is calculated as the variance of the spectral differences (decibel differences of the HRTF magnitudes) over frequency.

(1) $$\begin{array}{cc} {\mathrm{ISSD}}_{dir}\left(\theta ,\phi \right) & =\mathrm{var}(20\log_{10}\frac{|{\mathrm{HRTF}}_{1}\left(f,\theta ,\phi \right)|}{|{\mathrm{HRTF}}_{2}\left(f,\theta ,\phi \right)|})) \end{array}$$

(2) $$\begin{array}{cc} \mathrm{ISSD} & ={\mathrm{mean}}_{w}\left({\mathrm{ISSD}}_{dir}\right) \end{array}$$

The single value ISSD of an HRTF dataset is calculated as the weighted mean over all directions. The weights in this case are Voronoi-area weights to compensate for the denser spatial sampling at the poles compared to the horizontal plane resulting from the chosen equiangular sampling. The second metric used is the frequency-dependent Spherical Differences metric [18] that is calculated as the weighted standard deviation of all the spectral difference at a given frequency evaluated across all directions of the HRTF sphere:

(3) $$\mathrm{SD}\left(f\right)={\mathrm{std}}_{w}\left(20\log_{10}\frac{|{\mathrm{HRTF}}_{1}\left(f,\theta ,\phi \right)|}{|{\mathrm{HRTF}}_{2}\left(f,\theta ,\phi \right)|}\right)$$

**Figure 2:**
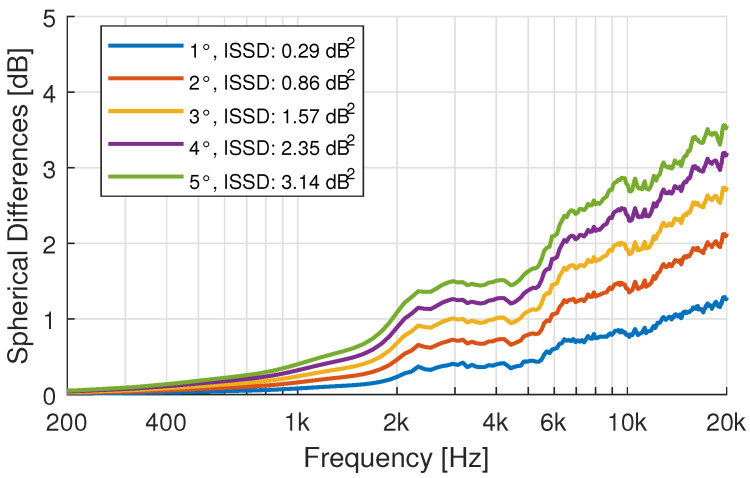
Spherical Differences: Effect of azimuth angle offset on the Spherical Differences and ISSD difference metrics of a 1 × 1-degree HRTF measurement of the KEMAR.

As a baseline for later comparisons, the effect of an azimuth-angle mismatch is evaluated in Figure 2. Here the simulated HRTF of participant 4 with a 1-degree resolution is compared to the same HRTF rotated along the z axis by an azimuth (ϕ) offset. The error increases with frequency and rotational offset due to the increased spatial complexity of the directivity at higher frequencies.

## 3. Results

### 3.1. 3D-Models: Mesh Resolution

**Figure 3:**
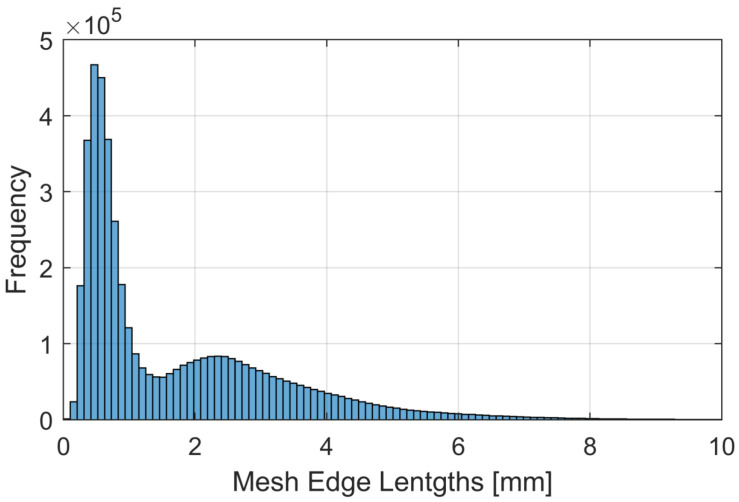
Histogram of the mesh edge lengths across all participants in the database.

The resulting 3D-models have an average edge length (AEL) of 1.7 mm, which is sufficient to simulate HRTFs using BEM simulations [59]. A histogram of the edge lengths of the provided meshes can be seen in Figure 3.

**Figure 4:**
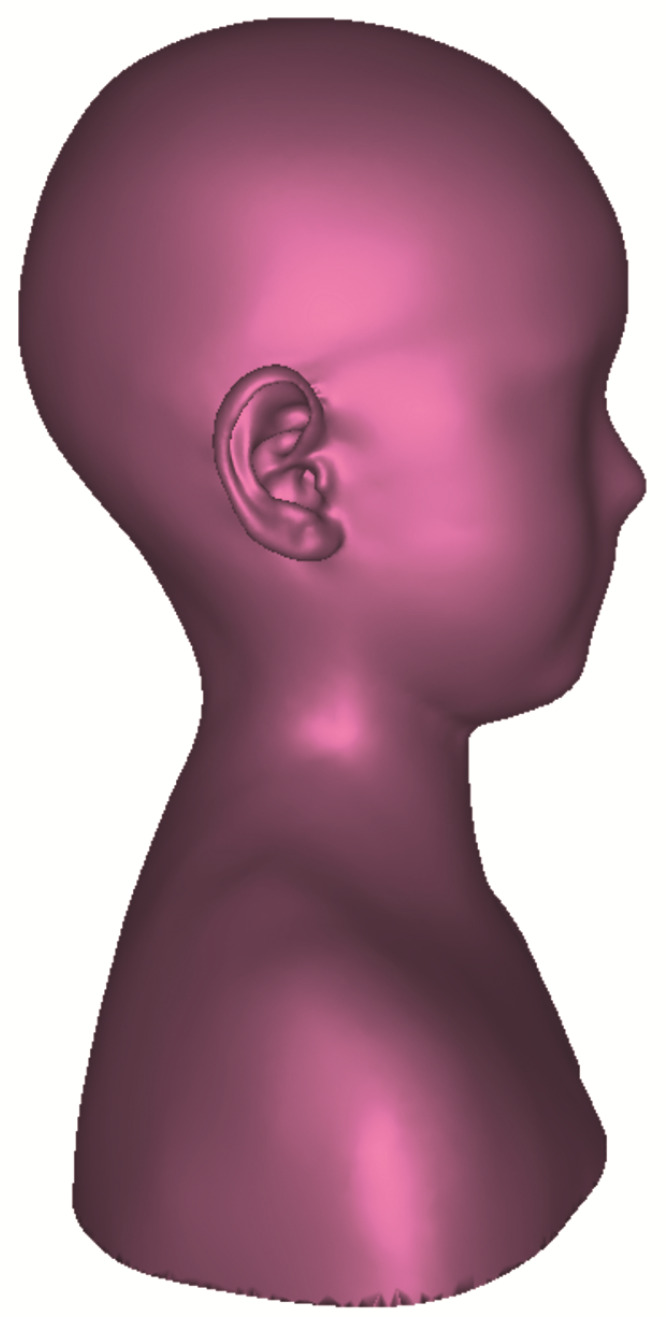
Exemplary anonymized 3D-surface mesh of one participant.

It shows a bimodal distribution resulting from the mesh reduction approach of keeping a high, sub-millimeter resolution for the pinnae while reducing the rest of the mesh, especially the torso strictly based on the curvature of the mesh while keeping the geometry accurate. All fine structure details that were not part of the pinnae (e.g., fine structures of the clothes and hair not covered by the cap) were removed and smoothed, as they contribute less to the HRTF characteristics. As an example, the resulting anonymized model of participant 4 can be seen in Figure 4.

### 3.2. 3D-Models: Anthropometric Measurements

Anthropometric measurements in accordance with the CIPIC database [46] were taken based on the 3D-models. Table 1 presents a statistical overview of a select number of these measurements. It is noticeable that the values can differ by a factor of 1.5 between the maximum and minimum values, which substantially affect the time and frequency characteristics of the corresponding HRTFs.

### 3.3. HRTF Measurement: Head Movement

To analyze the motion during the HRTF measurement, the calibrated tracking data of the center of the participant’s head was evaluated. Missing data-frames from short dropouts caused by the moving arc obscuring the vision of one of the motion tracking cameras were interpolated by the Optitrack system. The zero position and orientation of the rigid body were defined as the mean over the first 10 s of the measurement. Deviations from that zero value for all time samples within the duration of the HRTF measurement are presented in the following.

**Figure 5:**
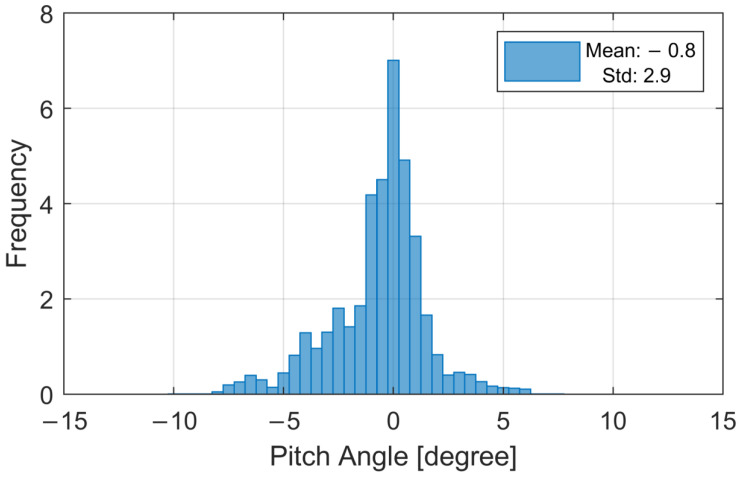
Pitch angle deviation during the HRTF measurement from all participants.

An evaluation of the pitch angle deviation, i.e., raising and lowering of the chin, presents a mean that is slightly negative at −0.8 degrees (c.f. Figure 5). This is a known observation present also in adult participants. The head gets heavy throughout the measurement duration, and the chin starts to dip down, even when focusing on a distant point at head height. The overall spread of the data indicates (std: 2.9 degrees) that the children generally were able to hold a steady gaze. However, their movement was more extensive than observations from adult participants. After outlier removal by median absolute deviation (MAD), these values improve to a mean of −0.5 degrees and a standard deviation of 1.6 degrees.

The overall translatory motion of the head during the measurement was evaluated as the Euclidean distance from the zero position. On average, the head moved by 0.82 cm with a standard deviation of 0.99 cm from the center position during the measurements. Again, this indicates, that the children were able to keep a steady head position with some outliers, who glanced at their parents or moved their head for other reasons. These movements were only of short duration before refocusing on the screen in front of them, thus affecting only a small number of measured directions.

### 3.4. HRTF Measurements: Comparison to the KEMAR Artificial Head

**Figure 6:**
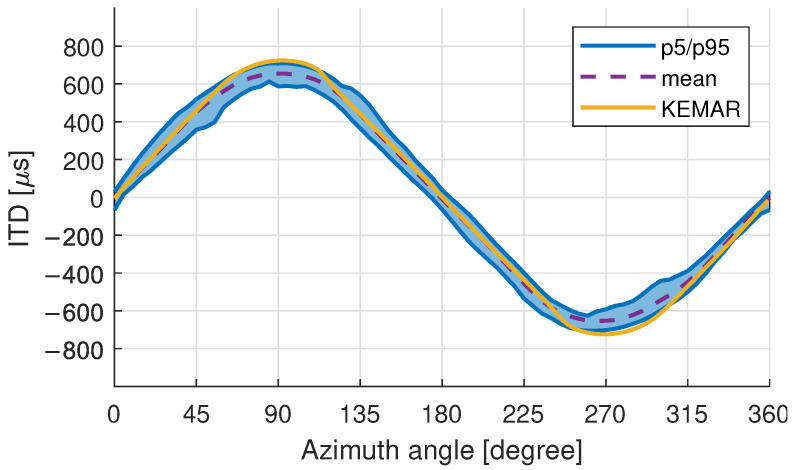
ITD data in the horizontal plane of children HRTF measurements compared to a KEMAR artificial head.

The KEMAR head and torso simulator [21] by GRAS (GRAS Sound & Vibration, Holte, Denmark) is an artificial head frequently used in acoustic and hearing aid research and as the HRTF in virtual environments for children (e.g., [34,37]). The HRTF of a GRAS 45BB-4 KEMAR (the measurement including a technical report is available for academic use from 10.18154/RWTH-2021-06298) [60] was compared to the measured child HRTFs of the database. The KEMAR was equipped with GRAS 40AO microphones positioned at the ear canal entrances without an ear canal simulator resulting in closed ear-canal measurement conditions comparable to the child-HRTFs. A comparison of the ITD between the KEMAR and children is shown in Figure 6. The mean ITD (in purple) along with the 5th and 95th percentile (in blue) of the child-database is compared to the KEMAR ITD (in yellow) in the horizontal plane. The ITD was calculated based on the phase-delay between 500 Hz and 1500 Hz [61].

**Figure 7:**
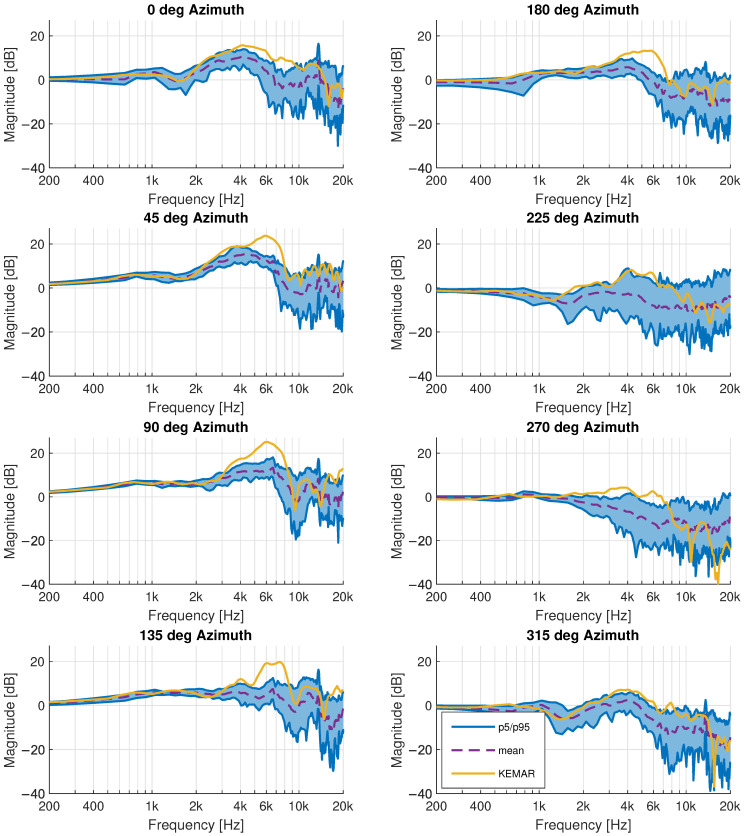
Horizontal plane HRTF comparison between the measured children dataset and the KEMAR artificial head. The 5th and 95th percentile (p5/p95) in the children database are shown in blue.

The maximum ITD value of the KEMAR is approximately 724 μs at 90-degree azimuth, the 95th percentile of the children HRTFs is 709 μs. This compares well with the head width of the KEMAR of 152 mm being close to the maximum and 95th percentile of the children database presented in Table 1. The mean child ITD has a maximum at 654 μs and a 5th percentile of 586 μs, resulting in a difference of 70 μs and 138 μs respectively. Previous studies found the just noticeable difference (JND) in ITDs varies between 18 μs [62] and 30 μs to 60 μs [63]. The differences between the children and the KEMAR are thus expected to be an audible. These JND values have after all been reported only for adults and have not been validated for children. A comparison of the HRTF magnitude for eight equiangular directions in the horizontal plane can be seen in Figure 7. As for the ITD, the magnitude response of the KEMAR is generally closer to the 95th percentile of the child HRTF database, especially at the dominant second pinna resonance (P2) around 5–6 kHz [64].

### 3.5. HRTF Comparison: Measurement and Simulation

**Figure 8:**
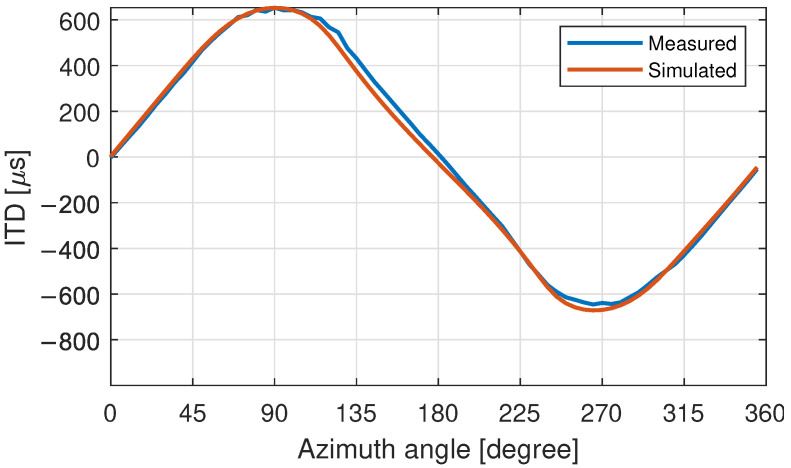
Comparison of measured and simulated ITD of participant 8.

**Figure 9:**
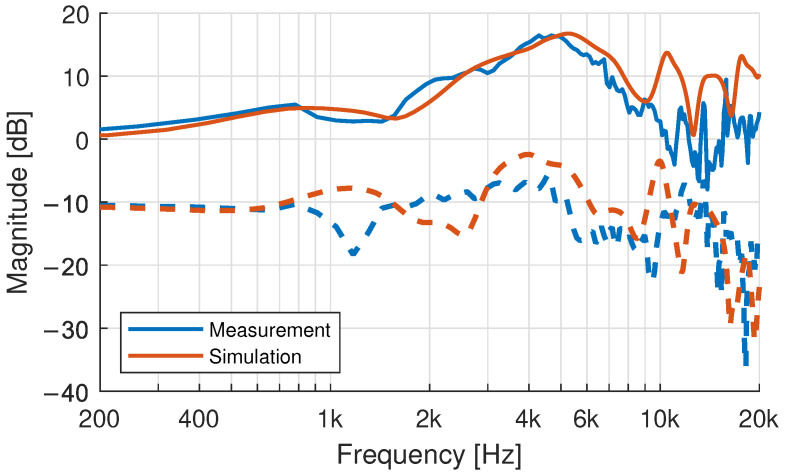
Exemplary comparison of measured and simulated magnitude spectra of a participant at 15-degree azimuth in the horizontal plane. The right ear transfer function (**dashed line**) is offset by −10 dB compared to the left (**solid line**).

In order to evaluate effects of the movement and the quality of the dataset, HRTFs were calculated based on the 3D-surface models using a BEM-simulation. 3D-models with longer torso sections were cut off approximately 20 cm below the top of the collarbone, to reduce computational efforts while retaining the influence of the shoulder reflections at low frequencies. As expected, the HRTF measurement and simulation give similar results as shown for the ITD in Figure 8 and HRTF magnitude in Figure 9. Some differences were expected as several simplifications needed to be made, to efficiently set up the models and solve the boundary-element problem. Surface impedances from clothes and the removal of hair in the simulations do affect the simulation accuracy [56,65], as does the size and placement of the excitation (i.e., the simulated microphones) [57].

Differences in posture between the scan and measurement led to HRTF differences especially at lower frequencies from mismatched shoulder/torso reflections. At higher frequencies, a spherical misalignment of the heads between the two datasets is a more significant error-factor. A static azimuth angle offset was compensated by ITD alignment (the zero crossing of the ITDs rising edge is set as the frontal direction) of the simulation result rounded to 1 degree. Other factors, such as dynamic movement or as constant pitch-angle offset, resulting from the participant looking a bit more up- or downwards, however, cannot be compensated that easily. The measured HRTFs are affected by a number of other factors [66], such as measurement noise e.g., from amplifiers and outside sound sources inherent in real- world acoustic measurements especially at the averted-ear HRTF measurements. Also, the SH-processing affects the accuracy of the HRTF, especially because only a spherical cap could be sampled due to the limitations of the room and speaker arc (no loudspeaker below −70-degree elevation) that needs to be considered through regularization (e.g., [67,68]).

**Figure 10:**
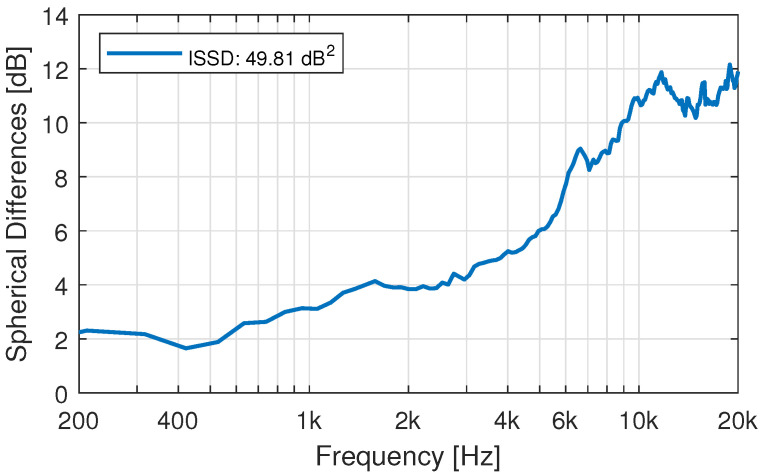
Spherical differences between measurement and simulation of participant 15.

While single directions can show a good agreement, the evaluation over the whole sphere, as shown in Figure 2, indicates that the above-mentioned effects do add to a substantial overall difference between measured and simulated HRTFs (c.f. Figure 10). The error introduced by the fast measurement system under ideal circumstances, i.e., when using an artificial head instead of a moving subject, accounts for spherical differences of about 4 dB at high frequencies [50].

### 3.6. HRTF Simulation: Effect of the Anonymization of the 3D-Models

**Figure 11:**
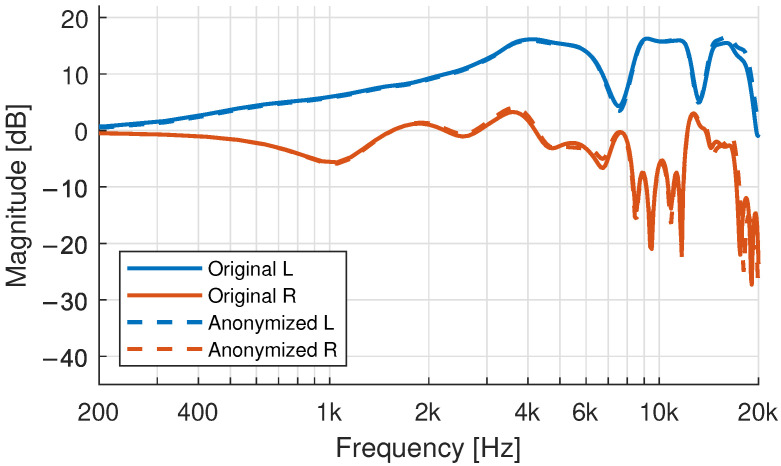
Comparison of simulated HRTFs of the non-anonymized (original) 3D-model and the published version with an anonymized face at 45 degrees azimuth in the horizontal plane.

**Figure 12:**
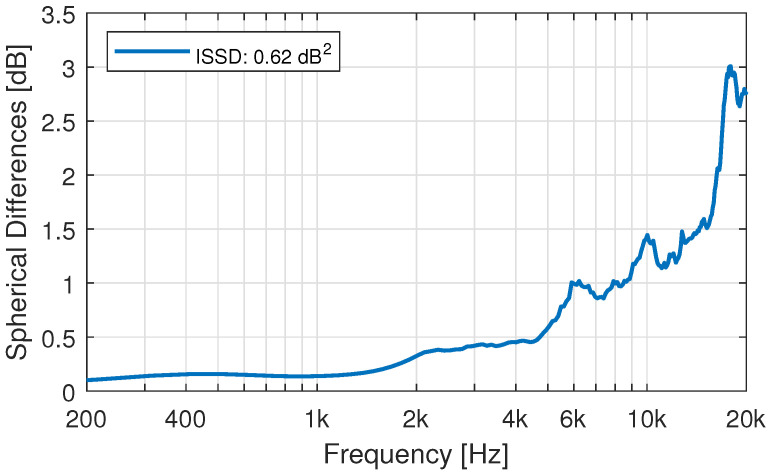
Spherical Differences between the simulated HRTF of the anonymized and the non-anonymized head and torso model of one exemplary participant.

The 3D-models have been anonymized by extensive surface smoothing of the face. To evaluate the effect of these geometry alterations on the simulated HRTF, BEM-simulations of the non-anonymized and anonymized 3D-model of an exemplary participant were compared. Figure 11 shows the effect on a single direction at 45-degrees azimuth in the horizontal plane. Only minimal differences can be seen below 16 kHz. At very high frequencies small differences between the two HRTFs can be observed. The same can be said about the spherical difference evaluation presented in Figure 12. The magnitude of the differences compares to an angular mismatch of 1–2 degrees. The mismatch shows a stronger bias towards high frequencies compared to Figure 2. The simulation mesh was optimized for simulations up to 20 kHz. These artifacts can thus already be the results of mesh-accuracy limitations [59].

## 4. Discussion

The quality of an HRTF measurement is affected by many factors [66]. For the presented data, especially the head movement and the influence of the fast measurement system are the most prominent. The influence of head movement during the measurements were known from pre-trials. Children are not able to stay perfectly still during long measurements, which is why a fast measurement system was chosen. The translatory motion of the head was kept to a zero mean with a 0.8 cm standard deviation. The rotary motion in the worst direction, the pitch angle (nodding movement), showed a mean of −0.8 degrees and standard deviation of 2.9 degrees. In comparison, adults show comparable head movements only in longer duration measurements for which rotary movements of up to 5 degrees (data from [69] evaluated in [69,70]) and standard deviations of up to 8 degrees [71] have been reported. The effect of head movement was estimated by Riederer [72] to be comparable to constant head rotation offset with general differences of 1 dB per degree offset angle. He reported the strongest effects at frequencies above 6 kHz in ipsilateral azimuth directions.

The post-processing needed due to the continued movement of the measurement loudspeakers also effect the measurement. Richter and Fels evaluated the same system used in this work and reported that the inaccuracies increase with measurement speed, especially towards higher frequencies. However, significant audible affect were not observed in a subjective evaluation at the chosen rotational speed [50]. Differences can especially be seen in the direct comparison of the measured HRTF with a simulated HRTF of the same participant based on a high-resolution 3D-scan of the same participant. The same is observed in repeated HRTF measurements of the same person, large differences of up to 30 dB at high frequencies [73]. Even round-robin measurements of the same artificial head in different labs showed differences exceeding 5 dB when using the artificial head microphone and almost 20 dB with alternate microphones, i.e., the microphones typically used by the institutions participating in the round robin experiment [74,75]. The 3D-models are also made publicly available with smoothed facial features. This method to anonymize the models has shown negligible effects on simulated HRTFs especially compared to the effects other modifications, such as neglecting the hair [56,65]. A comparison of the children’s HRTFs with a KEMAR artificial head found it is comparable only to the largest heads found in the collected data. A previous comparison of a prior version of the database with a different artificial head and an adult HRTF database [47] demonstrated that the artificial head [76] fell in between the adult and child in Terms of ITD [77]. An evaluation of the expected localization performance using a model by Baumgartner [78] in that study saw a slightly worst performance in the predicted quadrant and polar error rates of the children compared to adults, indicating that it is better suited for adults than children. These results found between the child and the artificial head HRTFs do result in actual perceptual differences, as the models used to estimate whether the difference are audible have also been developed with adult participants.

## 5. Conclusions

Virtual acoustic environments are a potent tool for the manipulation and evaluation of complex auditory scenes. Well matching HRTFs are essential for an authentic binaural presentation using headphones. However, HRTF measurements of children are challenging, as especially head movements during the measurements can cause inaccuracies. Movement during the presented measurements could be kept to a minimum through the use of a fast measurement system and a visual target for the children to focus on. A comparison of the measured HRTF data with the commonly used KEMAR indicates that the artificial head might not be appropriate when working with children. The children’s smaller head and pinna geometries lead to spectral and temporal differences in the HRTF, so that the KEMAR is not representative of an average transfer function for children under the age of ten. It rather represents only of the top percentile in this age group. This warrants a closer look at using child specific HRTFs in the binaural presentation of virtual acoustic environments as well as in other applications relying on HRTFs, such as hearing aid algorithms, video games or other hearing related research in the future. From the objective differences seen between the measured HRTFs and the KEMAR artificial head, perceptual implications on speech perception and other aspects can be expected. Yet the actual perceptual effects on children do need to be evaluated in future listening experiments.

## Figures and Tables

**Table 1:** Statistical summary of selected anthropometric measures in the database in [mm]. The shortnames refert to the definitions in the CIPIC database [46].

Dimension	min	p5	Mean	p95	max
x1: head width	114.4	133.5	142.6	150.7	153.0
d3: cavum concha height	14.1	14.8	17.25	18.8	20.9
d5: pinna height	51.8	52.0	55.6	60.1	63.6
d6: pinna width	26.7	27.2	30.8	35.8	36.3
d8 cavum concha depth	12.9	13.7	16.0	19.0	19.6
x12: shoulder width	274.2	283.3	322.7	365.4	371.8
x14: height	119	120.3	133.2	145.8	160.0

## Data Availability

The hrtf datasets presented in this publication including characterizations of the used transducers are openly available as part of the CHASAR database at https://doi.org/10.18154/RWTH-2021-06298, accessed on 16 December 2021.
